# Multimodality imaging of indolent B cell lymphoma from diagnosis to transformation: what every radiologist should know

**DOI:** 10.1186/s13244-019-0705-y

**Published:** 2019-02-22

**Authors:** Francesco Alessandrino, Pamela J. DiPiro, Jyothi P. Jagannathan, Gosangi Babina, Katherine M. Krajewski, Nikhil H. Ramaiya, Angela A. Giardino

**Affiliations:** 1 Department of Imaging, Dana Farber Cancer Institute, Harvard Medical School, 450 Brookline Avenue, Boston, MA 02215 USA; 2 Department of Radiology, Brigham and Women’s Hospital, Harvard Medical School, 75 Francis Street, Boston, MA 02115 USA; 3 Department of Radiology, UH Cleveland Medical Center, Case Western Reserve University, 11100 Euclid Ave, Cleveland, OH 44106 USA

**Keywords:** Transformation, Follicular lymphoma, Diffuse large-cell lymphoma, Chronic lymphocytic leukemia, Nodular-lymphocyte predominant Hodgkin’s lymphoma

## Abstract

Indolent B cell lymphomas are a group of lymphoid malignancies characterized by their potential to undergo histologic transformation to aggressive lymphomas. While different subtypes of indolent B cell lymphomas demonstrate specific clinical and imaging features, histologic transformation can be suspected on cross-sectional imaging when disproportionate lymph node enlargement or new focal lesions in extranodal organs are seen. On PET/CT, transformed indolent lymphoma may show new or increased nodal FDG avidity or new FDG-avid lesions in different organs. In this article, we will (1) review the imaging features of different subtypes of indolent B cell lymphomas, (2) discuss the imaging features of histologic transformation, and (3) propose a diagnostic algorithm for transformed indolent lymphoma. The purpose of this review is to familiarize radiologists with the spectrum of clinical and imaging features of indolent B cell lymphomas and to define the role of imaging in raising concern for transformation and in guiding biopsy for confirmation.

## Key points

- Indolent lymphomas can undergo histologic transformation to aggressive lymphomas
- Imaging findings of indolent and transformed lymphomas are presented
- Imaging can raise concern for transformation and guide biopsy for confirmation
- An algorithm for management of transformed indolent lymphoma is proposed

## Introduction

Lymphoma encompasses a heterogeneous group of lymphoid malignancies accounting for 4% of all cancers diagnosed in USA, with around 20,000 estimated deaths in 2016 [1]. First described in 1666 by Marcello Malpighi’s “De viscerum structura exercitatio anatomica”, lymphomas have been classified and reclassified throughout the last 50 years, with each classification reflecting the biological knowledge, the immunologic understanding, and therapeutic trends of the moment [2–6]. While the most recent classification from the World Health Organization divides lymphoid malignancies according to their immunological phenotype (into mature B cell, T and NK cell neoplasms, Hodgkin lymphoma (HL), post-transplant lymphoproliferative disorders (PTLD), histiocytic and dendritic cell neoplasms)), in clinical practice lymphomas are divided into indolent or low-grade lymphoma and aggressive or high-grade lymphoma [6–8]. The term “indolent lymphoma” was first introduced in 1974 to describe a group of lymphoid malignancies which share clinical and prognostic features of indolent clinical course and relative resistance to therapy [8].

Indolent lymphomas may undergo histologic transformation (HT), which is defined as evolution from indolent, low-grade lymphoma to aggressive, high-grade lymphoma, by means of genetic mutations with corresponding modifications in histologic architecture, clinical behavior, and prognosis of the disease [9, 10]. Clinically, transformed indolent lymphoma (TIL) presents with new systemic or “B” symptoms (unexplained weight loss, fever, and profuse night sweating), rapid or discordant nodal growth, new involvement of extranodal sites, rising lactate dehydrogenase (LDH), and hypercalcemia [9, 11]. Imaging has a crucial role in recognizing TIL: it helps to identify transformed lymph nodes or extranodal site involvement to guide biopsy, or can raise concern for transformation before clinically evident. In addition, imaging may allow differentiation between HT and recurrent/progressive indolent lymphoma or secondary malignancies, with change in patient management and impact on patient prognosis [9, 11–14]. Transformation commonly occur to diffuse large B cell lymphoma (DLBCL), less commonly to other types of aggressive lymphoma, including Burkitt and T cell/histiocyte-rich B cell lymphoma (TCRBCL) [9–11, 15, 16]. Recognizing and diagnose transformation is crucial since prognosis and management of indolent lymphoma and its transformed counterpart highly differs. While follicular lymphoma (FL), the most common indolent lymphoma, shows median survival of 14 years, after transformation survival drops to 1–2 years [9, 11, 17]. In addition, while treatment and follow up care of indolent lymphoma is based on the stage and subtype of disease, treatment and surveillance of transformed indolent lymphoma (TIL) is individualized, as currently there are no randomized studies in the modern era to guide practice [11, 12].

In this paper, after a brief introduction on indolent lymphomas, we will present the clinical and imaging features of most common subtypes of indolent lymphomas, we will discuss the imaging features of HT for the different subtypes of indolent lymphoma and its differential diagnosis, and eventually we will propose a imaging algorithm for diagnosis and management of TIL, so to provide the radiologist with the appropriate clinical tools to recognize indolent lymphoma from diagnosis to transformation.

### Indolent lymphoma: general considerations

Indolent lymphomas are characterized by a long course of disease, with death occurring years after diagnosis [18]. Diagnosis is made through excisional—or core-needle when not feasible—biopsy of the lymph node or extra nodal tissue involved, and based on morphologic, immunophenotypic, and genetic data [6]. Subsequently, patients should undergo clinical, laboratory, imaging evaluation including PET-CT or CT to stage the disease according to the modified Ann Arbor staging system based on lymph node involvement and the presence of B symptoms in case of HL [19]. Once diagnosis and staging has been defined, the first step in management is to decide when to start therapy, and eventually which therapy regimen is appropriate [20]. Prognostic scores may also aid in decision, including the Follicular Lymphoma International Prognostic Index (FLIPI) score, which is based on patient’s stage, clinical and laboratory findings, and predicts overall survival rate in patients with FL [18, 21]. As a general rule, watchful waiting is advisable in asymptomatic patients with low-grade disease, although rituximab only has been proposed by some authors, with only limited increase in progression-free survival [22]. In limited stage disease, radiation therapy is generally used and rituximab-added chemotherapeutic regimens for more advanced stage or high-grade disease. Hematopoietic stem cell or bone marrow transplant is reserved in selected cases [10, 23]. Once decision on therapy has been made, follow up care should be set and varies according to lymphoma subtype and likelihood of progression, relapse, or transformation [11].

After a variable number of years of follow-up, generally with transient and incomplete response to therapy, death may occur due to disease progression, histologic transformation, or for unrelated causes [1, 18, 22].

Management and treatment of the first two events, progression and HT, is challenging, as these events are associated with high death rates [18]. Generally, treatment is individualized, depending on patient status and prior therapies [24, 25]. In case of TIL, the R-CHOP regimen (rituximab, cyclophosphamide, hydroxydaunorubicin, oncovin, and prednisone) is recommended in patients with no prior treatment, radiotherapy, or non R-CHOP chemotherapy regimens [11, 24]. In patients with recurrent indolent lymphoma previously treated with R-CHOP, autologous stem cell transplant has been proposed [24].

### Clinical and imaging features of indolent lymphoma

The most common subtypes of indolent lymphomas undergoing HT are FL, chronic lymphocytic leukemia/small lymphocytic lymphoma (CLL/SLL), marginal zone lymphomas (MZL), Waldenstrom macroglobulinemia/lymphoplasmacytic lymphoma (WM/LPL), and nodular lymphocyte-predominant Hodgkin lymphoma (NLPHL) [9]. These subtypes represent the 96.5% of the indolent lymphomas [1]. Their clinical and imaging characteristics are presented in Table 1.

**Table 1:** Clinical and imaging characteristics of B cell indolent lymphomas undergoing histologic transformation

Subtype	Epidemiology	Clinical presentation	Imaging features	1/10 year risk of HT
FL	60 years old	Asymptomatic adenopathy (waxing and waning)	Multiple, deep non-obstructive adenopathy	3%/30%
	M > F	Signs of extranodal involvement	Splenomegaly or focal splenic lesions	
			Extranodal involvement: organomegaly or focal lesions	
			Bone marrow, liver, lungs, CNS (more common)	
			Thyroid, parotid, breast, testis, skin (less common)	
			FDG avidity: 91–100%	
CLL/SLL	71 years old	Asymptomatic lymphocytosis^a^	Adenopathy, splenomegaly,hepatomegaly	0.5–1%/16%
	Increases with age	Peripheral adenopathy	Heterogeneous bone marrow infiltration (MRI)	
	Rare < 40 years old	Splenomegaly	Brain and meningeal enhancement (MRI)	
		Anemia, bleeding, infections (cytopenia)	FDG avidity: 73% (high avidity–shorter survival)	
MZL	69 years old			0.5%/10%
MALT lymphoma		Organ specific symptoms	Adnexa oculi: enhancing issue infiltrating ocular appendages	
		Association:	Lung: lung nodules, consolidations, reticulation, peribronchial infiltrates	
		Helicobacter pylori infection	Gastrointestinal: smooth, polipoid or infiltrative lesions	
		Hashimoto thyroiditis		
		Clamydia Psittaci infection		
Splenic MZL		Splenomegaly, cytopenia	Single or multiple focal splenic lesions or splenomegaly	
Nodal MZL		Adenopathy	Adenopathy	
			FDG avidity: 49% (Ocular)–95% (Bronchial)	
WM/LPL	60 years old	Recurrent infections, easy bruising	Bone marrow involvement (MRI)	0.5%/2.4%
		Headache, Blurry vision	Diffuse: bones iso or hypointense to muscle	
		Neuropathy	Variegated: multiple enhancing foci in bone marrow	
		Organomegaly, adenopathy	+ Fractures	
			Lung, pleura, skin, liver involvement	
			CNS involvement (Bing-Neel syndrome)	
			T2 hyperintense hyperenhancing periventricular/subcortical foci	
			Meningeal or spinal enhancement	
			Adenopathy	
			FDG avidity: 73%	
NLPHL	Bimodal (childhood–4th decade)	Adenopathy	Adenopathy, splenomegaly, splenic lesions	0.73%/10%
	Rare (500 cases/year in USA)			

*HT* histologic transformation, *FL* follicular lymphoma, *CLL*/*SLL* chronic lymphocytic leukemia/small lymphocytic lymphoma, *MZL* marginal zone lymphoma, *MALT* mucosa associated lymphoid tissue, *WM*/*LPL* Waldenstrom macroglobulinemia/lymphoplasmacytic lymphoma; *NLPHL* nodular lymphocyte-predominant Hodgkin lymphoma; *CNS* central nervous system

^a^Defined as absolute lymphocyte count greater than 5000 cells/μL

Mycosis fungoides, a rare indolent T cell lymphoma which can transform to large T cell lymphoma, presents mostly with cutaneous lesions, will not be reviewed in this paper [1, 26].

#### Follicular lymphoma

Follicular lymphoma is the most common type of indolent B cell non-Hodgkin lymphoma (NHL), originating from centroblasts and centrocytes of germinal centers of the lymph nodes, the spleen, or the bone marrow and is characterized by several genetic mutations including the BCL2 translocation [11, 27, 28]. Follicular lymphoma is graded according to the number of centroblasts present at high-power field (HPF) histologic examination, from grade 1, with 0–5 centroblasts per HPF, to grade 3, with more than 15 centroblasts per HPF [27]. More than 90% of the diagnosed FL are grade 1 and 2 [27].

**Fig. 1:**
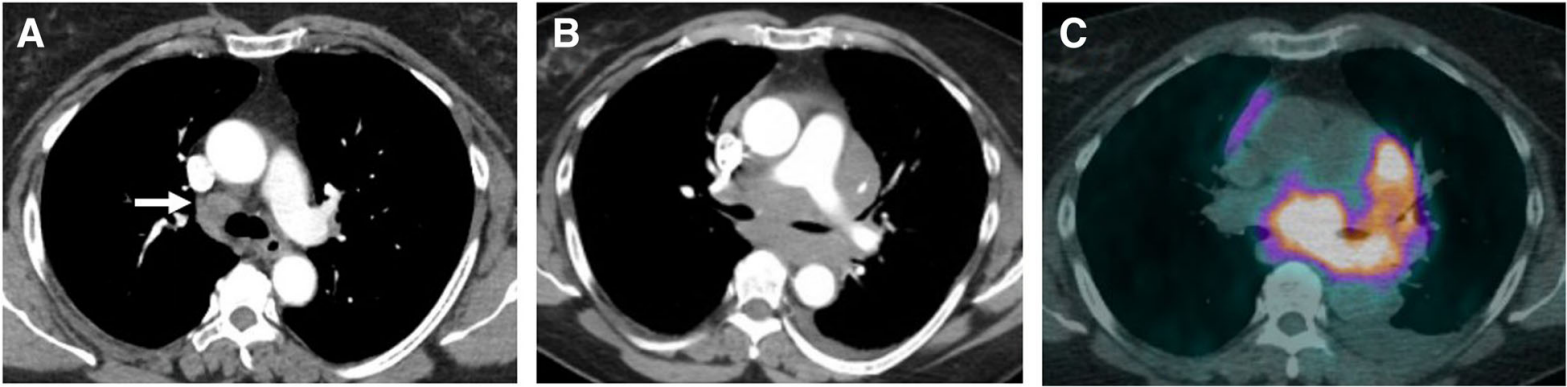
A 60-year-old woman with grade I follicular lymphoma on rituxan, with new onset shortness of breath and atrial fibrillation. **a** Axial CT image of the chest acquired during arterial phase 1 year before onset of new symptoms shows mildly prominent mediastinal lymph nodes (arrow). **b** Axial CT image acquired during arterial phase at time of symptoms shows a large amorphous mediastinal mass surrounding the distal trachea and the pulmonary artery. **c** Axial PET/CT fused image of the chest acquired at time of symptoms shows avid FDG uptake of the mass, with SUVmax 20.1. The lesion was biopsied and showed grade III follicular lymphoma

On cross-sectional imaging, FL presents with multiple, deep, non-contiguous enlarged lymph nodes, homogeneously enhancing on CT or MR (Fig. 1) [29]. Intra-abdominal adenopathy in general does not cause gastrointestinal or genitourinary symptoms [10]. The “sandwich sign,” has been described in patients with mesenteric large confluent adenopathy on both sides of mesenteric vessels, with the nodal masses representing the buns and the vessels resembling the sandwich filling, giving the appearance of a hamburger [30, 31]. An increased number of lymph nodes should raise suspicion for early stage of FL. FL can present also with extranodal involvement presenting with organomegaly or focal lesions [10, 29]. The most common extranodal sites involved are the bone marrow, liver, lungs, and central nervous system, whereas involvement of the thyroid, parotid gland, breast, testis, orbits, skin, and subcutaneous tissues is unusual. Splenic involvement can be in the form of splenomegaly and FDG-avid lesions on PET/CT, T2-hyperintense homogeneously enhancing lesions on MR or hypodense focal lesions on CT [29].

On PET/CT, FL is reported as FDG avid in 91–100% of cases, in general with low avidity, depending on the histologic grade of FL [19, 20, 32–34]. Various studies compared PET/CT with CT for FL staging, showing that in up to one third of cases, PET/CT alters the stage of FL, with consequences in patient management [12].

#### Chronic lymphocytic leukemia/small lymphocytic lymphoma

Chronic lymphocytic leukemia/small lymphocytic lymphoma represent a spectrum of disease ranging from a pure bone marrow and blood disease (CLL) to pure extramedullary disease (SLL), in which small mutated small lymphocytes undergo uncontrolled proliferation. When HT occurs in CLL, it is termed Richter transformation. This occurs with a 0.5–1%-year rate, with 16% probability of transformation at 10 years [35].

**Fig. 2:**
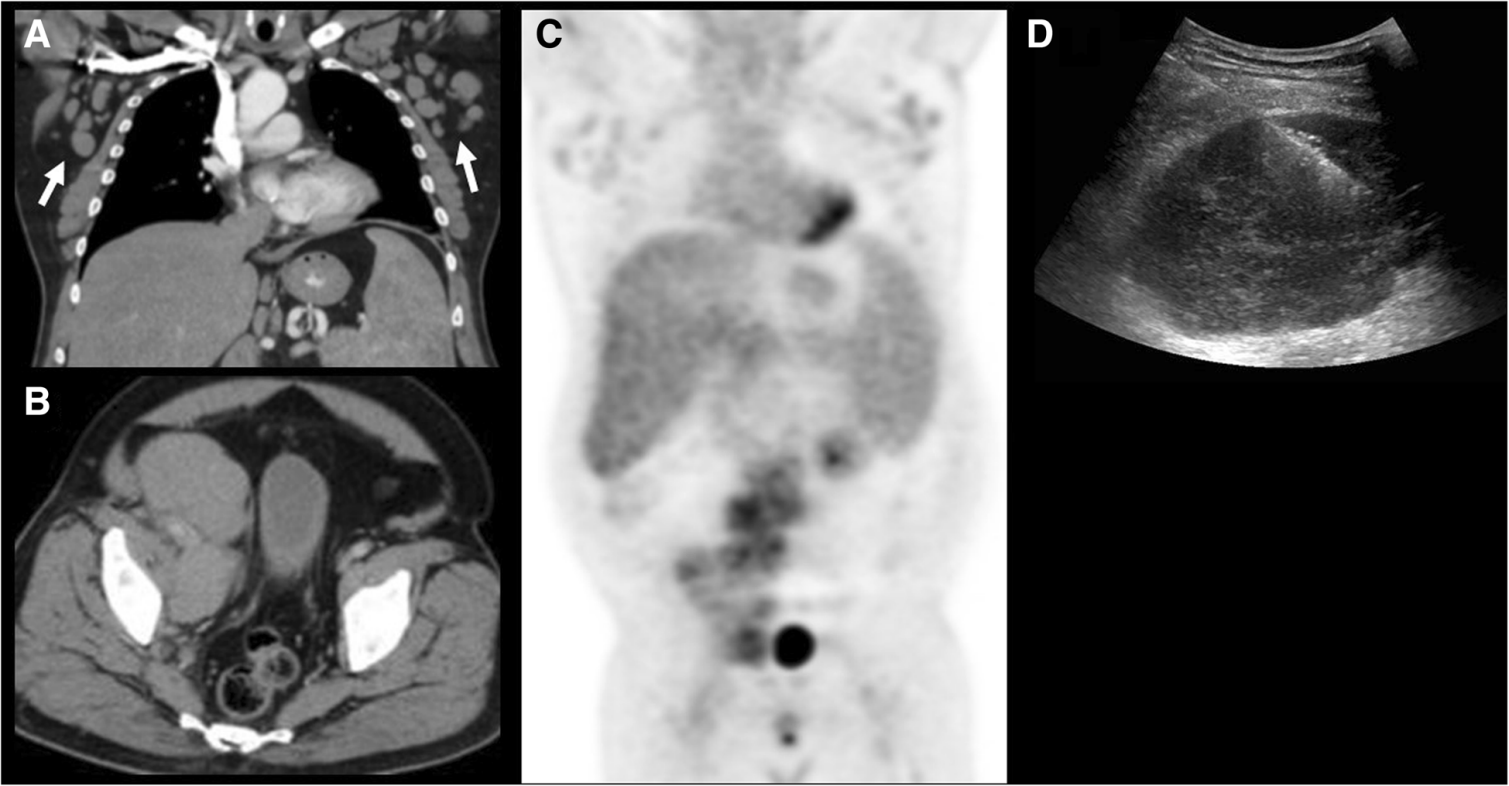
A 59-year-old man with chronic lymphocytic leukemia presenting with new onset night sweats, fatigue and left upper quadrant pain. **a** Coronal reconstructed and axial (**b**) CT images acquired during portal venous phase at the time of symptoms shows mildly enlarged axillary lymph nodes (arrows) and right pelvic adenopathy. **c** PET image demonstrated FDG-avidity of the pelvic lymph node. **d** Ultrasound guided biopsy of the right pelvic adenopathy demonstrated histologic transformation to EBV-positive Hodgkin lymphoma

Imaging findings of CLL/SLL include adenopathy (defined as lymph nodes with short-axis diameter > 10 mm, or as the presence of multiple small nodes in a single region) splenomegaly, hepatomegaly, and various degrees of bone marrow infiltration (Fig. 2) [36–39]. Brain parenchymal and meningeal involvement has also been reported in 4% of cases. This can be evaluated with contrast-enhanced MRI, showing variable degree of abnormal parenchymal and meningeal enhancement [40]. Regarding PET/CT, on a recent study on 526 patients with CLL, FDG avidity at diagnosis was observed on 384 (73%) cases, with high avidity in 120 (23%) cases. In this study, high FDG avidity was associated with shorter survival [41].

#### Marginal zone lymphoma

Marginal zone lymphoma represents a subset of lymphoma arising from the marginal zone of the secondary follicle [42]. According to the site of involvement, MZL has been classified in mucosa-associated lymphoid tissue (MALT) lymphoma, the most common subtype, splenic lymphoma, and nodal lymphoma [4, 43]. Histologic transformation occurs with a 0.5%-year rate, with 10% probability of transformation at 10 years [44].

**Fig. 3:**
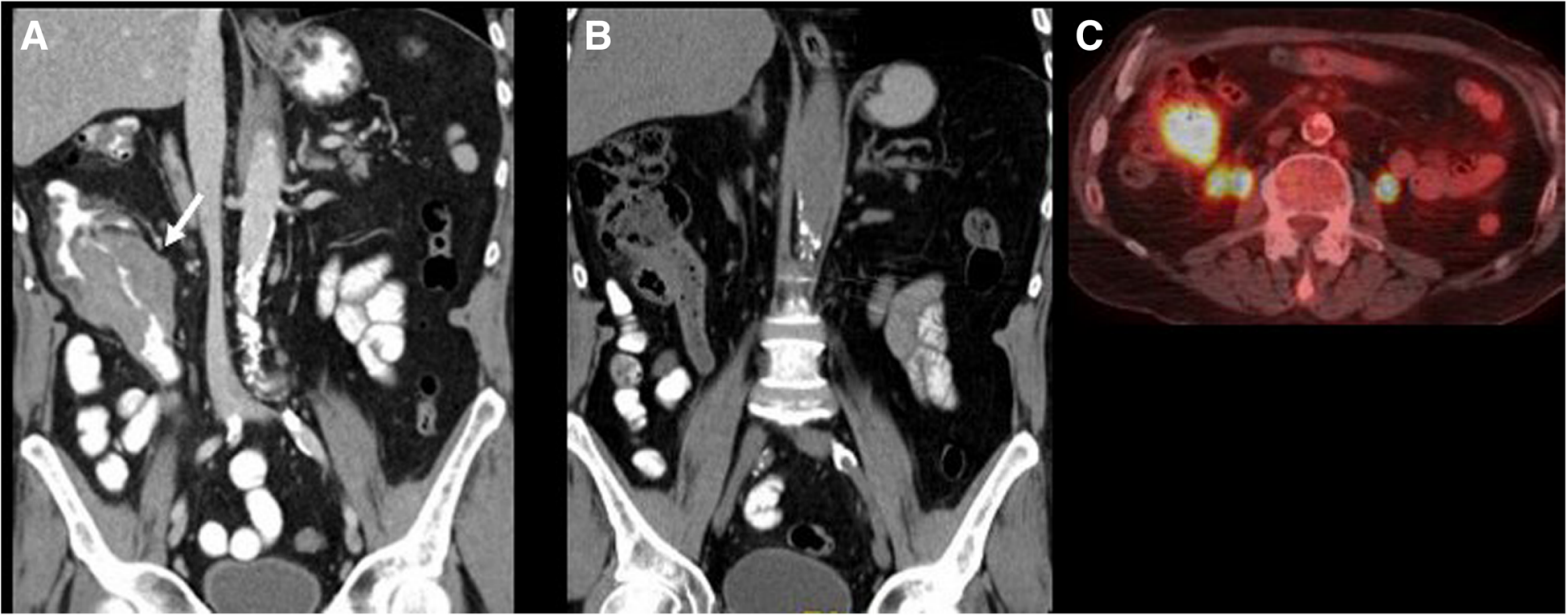
A 65-year-old man with history of MALT lymphoma treated with rituximab with new onset fever and fatigue. **a** Coronal reconstructed CT image acquired during portal venous phase at time of diagnosis shows a large mass at the ascending colon (arrow). Biopsy of the mass demonstrated MALT lymphoma. **b** Coronal reconstructed CT image after 6 months of treatment shows resolution of the mass. **c** PET/CT axial fused image at new onset of symptoms shows intense focal FDG uptake in the region of the ileocecal valve with SUVmax 15.9. Biopsy of the mass showed histologic transformation to diffuse large B cell lymphoma

Regarding imaging features, these depend on the location and subtype of MZL. MALT lymphoma of the ocular appendages (*adnexa oculi*) shows homogeneously enhancing T2-hyperintense or hypoattenuating soft tissue infiltrating the adnexa on CT or MRI. MALT lymphoma of the lung shows nodules, reticulations, consolidations, and peribronchial infiltrates [43, 45]. Imaging findings of gastrointestinal tract MALT lymphoma include smooth polypoid or infiltrative lesions, with rare gastrointestinal obstruction (Fig. 3) [45]. Splenic MZL demonstrates variable spleen involvement in the form of single or multiple focal lesions, splenomegaly, or miliary lesions (smaller than 0.5 cm) [46]. Sensitivity of PET/CT for MZL diagnosis ranges from 49 to 95%, depending on MZL subtype and localization. A recent meta-analysis showed pooled sensitivity of PET/CT of 49% for diagnosis of ocular and 95% for diagnosis of bronchial MALT lymphoma [47].

#### Waldenstrom macroglobulinemia/lymphoplasmocytic lymphoma

Waldenstrom macroglobulinemia/lymphoplasmocytic lymphoma (WM/LPL) is a B cell neoplasm in which malignant lymphocytes share morphologic characteristics with mature plasma cells [48]. When an IgM paraprotein is produced and detectable in the setting of bone marrow lymphoplasmacytic infiltration, the disease is typically referred to as WM, otherwise the disease is defined LPL [48, 49]. Histologic transformation occurs with a 0.5%-year rate, with a 2.4% probability of transformation at 10 years [50].

**Fig. 4:**
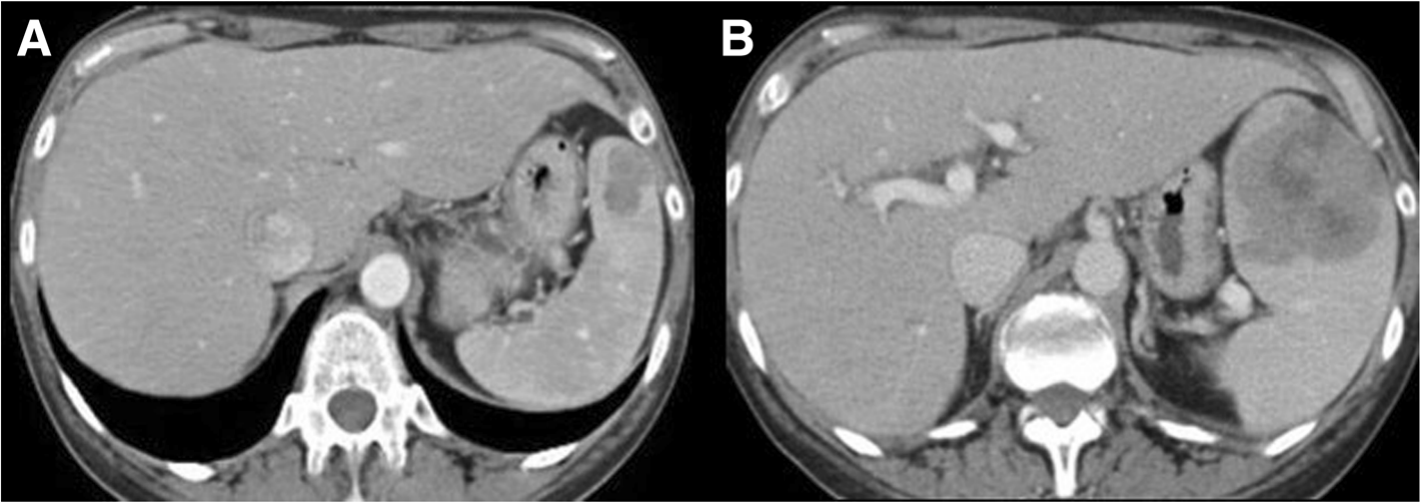
A 65-year-old woman with Waldenstrom macroglobulinemia treated with chemotherapy and hematopoietic stem cell transplant and new onset fever, neutropenia, and increased LDH. **a** Axial CT image acquired during portal venous phase six months before the onset of new symptoms shows a hypodense lesion in the spleen. **b** Axial CT image acquired during portal venous phase at the time of symptom onset shows increased size and decreased density of the splenic lesion. Biopsy of the lesion confirmed histologic transformation to diffuse large B cell lymphoma

Cross-sectional imaging findings include bone marrow involvement, adenopathy, extranodal involvement, or splenic lesions or splenomegaly (Fig. 4) [10, 51, 52]. Bone marrow abnormalities are seen on MRI in 90% of patients, according to a single-center study on 23 patients, in two forms: a diffuse or a variegated pattern [50]. In the diffuse pattern, the vertebral bones are diffusely iso- or hypointense to the adjacent paravertebral muscle, while the variegated pattern reveals innumerable tiny foci of marrow replacement scattered throughout the marrow, with various degrees of enhancement [51]. Vertebral body compression fractures can also be appreciated [51]. Extranodal sites of involvement were lungs, pleura, skin, liver, and bowel [52–54]. In addition, CNS involvement of WM (Bing-Neel syndrome) has been described in literature, with T2 hyperintense enhancing periventricular and subcortical lesions with variable diffusion restriction and associated meningeal enhancement on MRI [55, 56]. In addition, leptomeningeal or medullary enhancement can be seen in case of optic nerve or spinal cord involvement [56]. In a study on 35 patients with WM, FDG-PET/CT positivity was seen in 77% of cases [54]. PET/CT was found to be more sensitive in assessing response to treatment when compared to CT [57].

#### Nodular lymphocyte-predominant Hodgkin lymphoma

Nodular lymphocyte-predominant Hodgkin lymphoma is a rare subtype of HL characterized by the presence of scattered large hystiocytic and lymphocytic cells often referred as “popcorn” cells due to the multi-lobated or folded appearance of the nucleus [58]. Its clinical behavior is similar to indolent lymphoma, with slow growth, high rate of recurrence, and possibility of transformation [59–61]. A recent study on 222 patients with NLPHL reported a 0.73%-year rate of HT, with 10% probability of transformation at 10 years [58]. An increased rate of HT has been observed in patients with advanced stage and with intra-abdominal and/or spleen involvement at diagnosis [15, 58].

CT reveals adenopathy, more commonly supradiaphragmatic, in particular axillary or cervical, and spleen involvement [62, 63]. MRI can be helpful in assessing bone marrow involvement [64]. PET/CT shows FDG-avidity of the spleen, bone marrow, lymph nodes with sensitivity close to 100%, as reported in two studies on 31 and 35 patients [62, 63].

### Imaging features of transformation

**Fig. 5:**
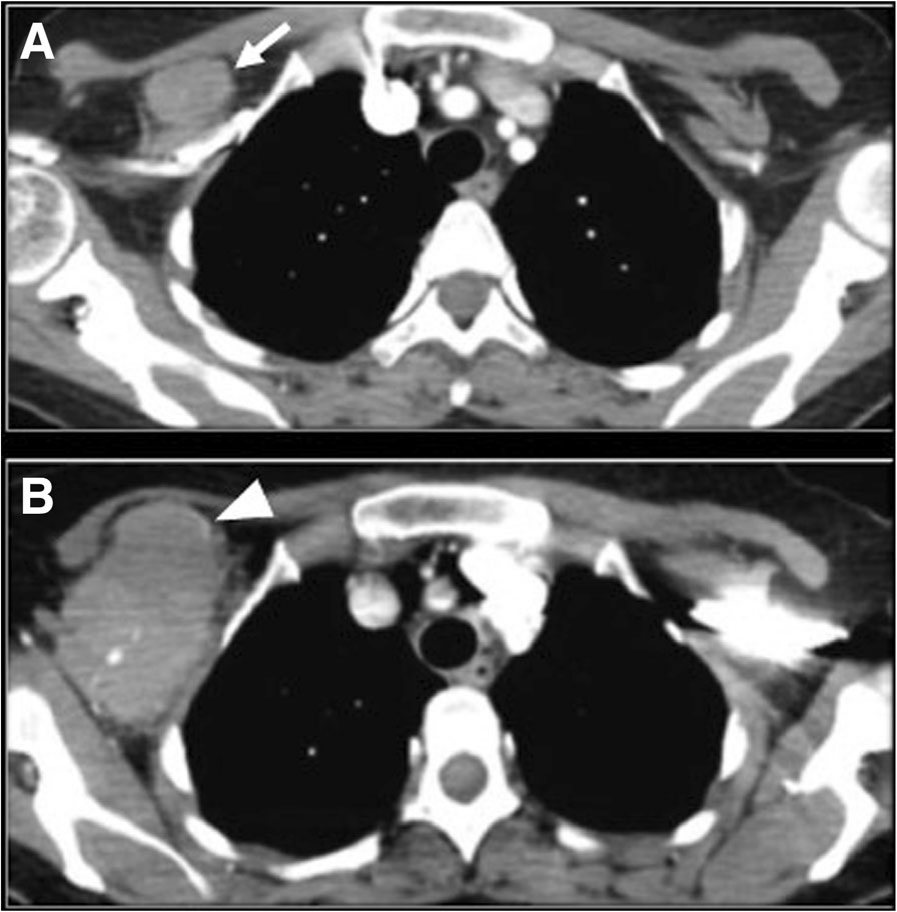
A 48-year-old woman with grade I follicular lymphoma treated with rituximab and increased LDH and right arm swelling. **a** Axial CT image acquired during portal venous phase 6 months before the onset of new symptoms shows a right retropectoral adenopathy (arrow). **b** Axial CT image acquired during portal venous phase at the time of increased LDH shows increased size and areas of decreased density of the retropectoral adenopathy (arrowhead). Biopsy of the lesion shows histologic transformation to diffuse large B cell lymphoma, with areas of necrosis

Histologic transformation of lymphoma can occur in lymph nodes, the spleen, or in extranodal locations [62, 65–67]. Nodal transformation can be suspected when disproportionate lymph node enlargement is noted on CT, MR, or US. Nodal enlargement can be localized to a single node; regional, when lymph nodes in a nodal station are increased in size; diffuse, when multiple nodal stations are involved (Fig. 2). In addition, transformed lymph nodes may show areas of decreased density on CT, reflecting areas of necrosis (Fig. 5), a finding that is extremely uncommon in uncomplicated indolent lymphoma [65]. On PET/CT, transformed lymph nodes show higher FDG-avidity with increased SUVmax when compared to other non-transformed nodes in the same patient (Fig. 2), or in case of diffuse HT, increased FDG avidity and SUVmax compared to prior scans, with different cut-offs depending on indolent lymphoma subtypes [62, 65–69]. In cases of spleen involvement, CT shows new or increased focal hypodense lesions (Fig. 4) or new or increased splenomegaly, which is also reflected on PET/CT by diffuse increase in FDG avidity or focal FDG avid lesions in the spleen [62].

**Fig. 6:**
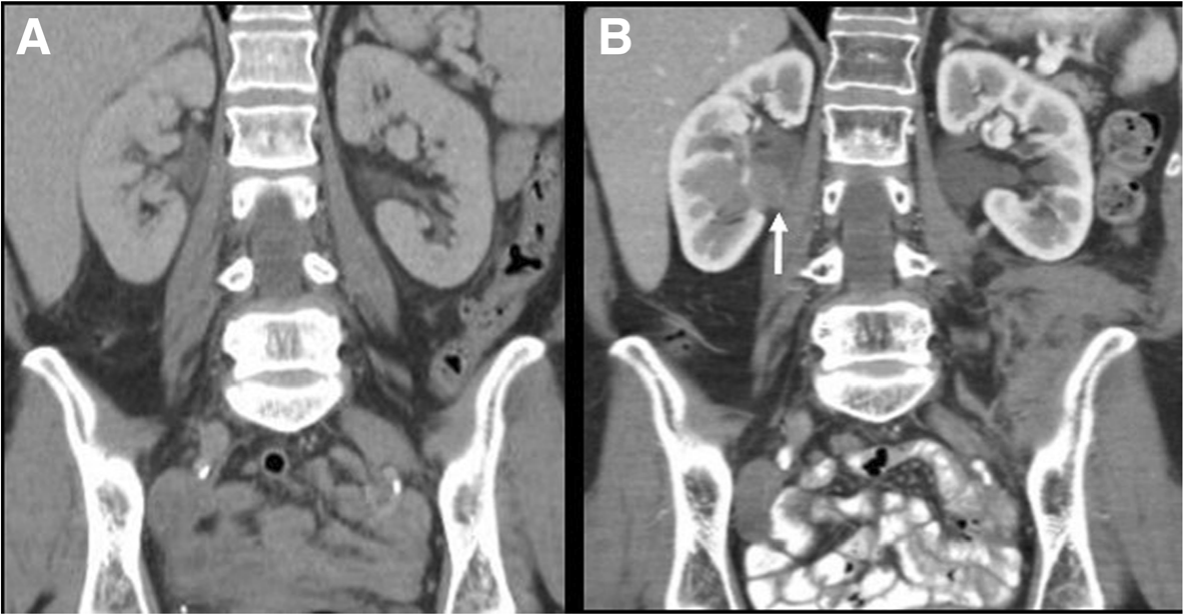
A 59-year-old woman with follicular lymphoma presenting with acute abdominal pain. **a** Coronal reconstructed CT image acquired during portal venous phase 6 months before the onset of new symptom shows unremarkable appearance of the abdomen. **b** Axial CT image acquired during late arterial phase at the time of symptom onset shows a mass within the right renal pelvis (arrow). Biopsy of the lesion shows histologic transformation to diffuse large B cell lymphoma

**Fig. 7:**
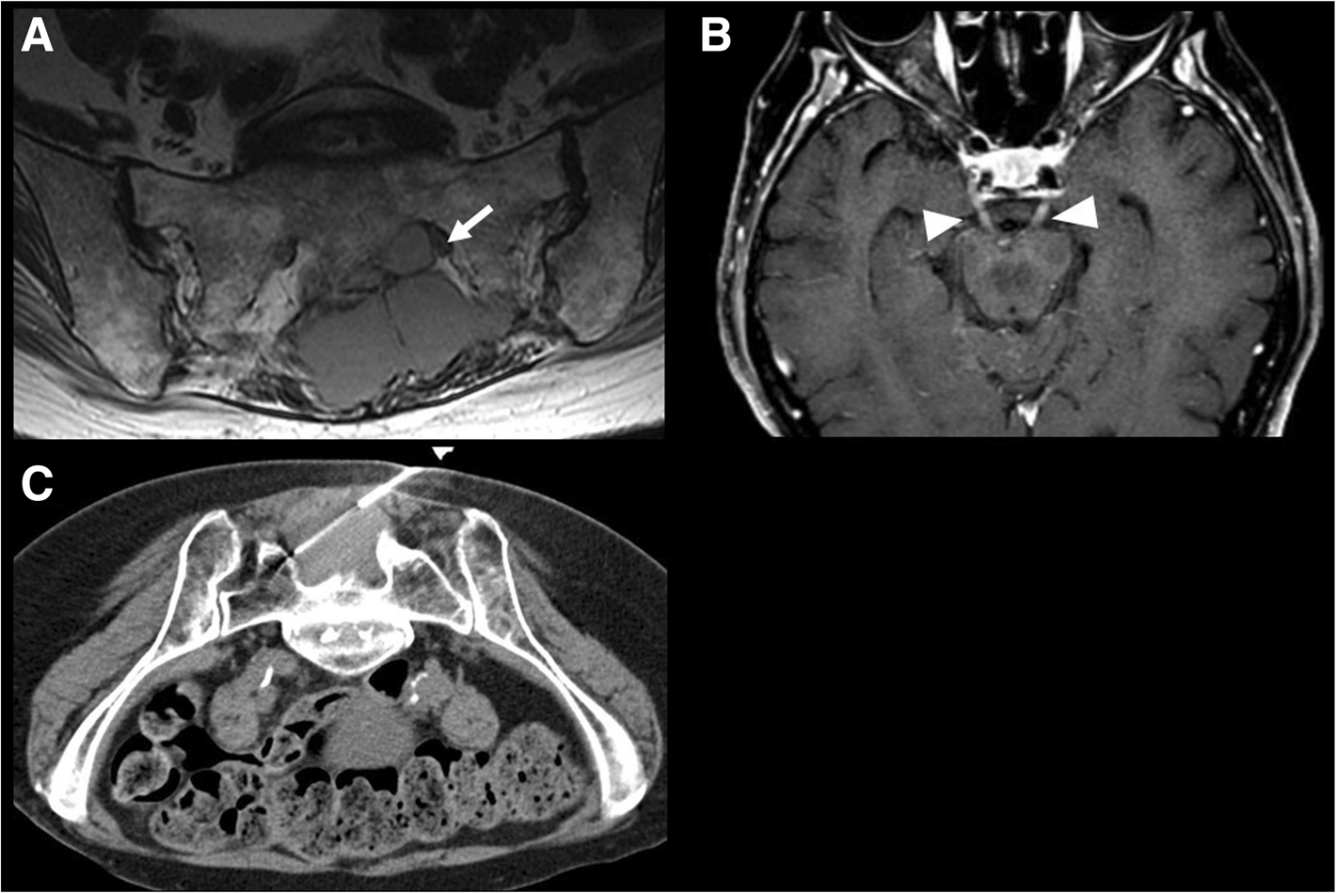
A 84-year-old woman with history of nodal marginal zone lymphoma treated with cyclophosphamide, hydroxydaunorubicin, oncovin, and prednisone, presenting with new onset back pain and acute diplopia. **a** T2-weighted axial image showed a large mildly hyperintense mass in the sacrum extending to the left sacral ala and into the left S1 foramen (arrow). **b** Axial T1-weighted images of the brain showed diffuse enhancement of the bilateral III cranial nerves (arrowheads). **c** CT-guided biopsy of the sacral mass demonstrated histologic transformation to diffuse large B cell lymphoma

Extranodal transformation may manifest with new focal lesions in various organs, which can present as hypodense homogeneous lesions in solid organs on CT, or in case of hollow viscera, new or increased wall thickening, such as bowel or ureteral wall thickening (Fig. 6). MRI can be useful in case of suspected brain or bone marrow transformation, showing new bone marrow lesions, new areas of enhancement in the brain parenchyma, or new meningeal or cranial nerve enhancement (Fig. 7). PET/CT shows new or increased FDG avidity of the involved organs (Fig. 3). Transformation can occur in site of prior involvement or at different extranodal sites (Fig. 3).

In addition, it is worth mentioning that since indolent lymphomas are composed of multiple subpopulations with distinct mutations, multiple transformations can occur in the same patient with low-grade disease, sometimes either simultaneously or sequentially [11].

Regarding different types of TIL, most studies focus on FL, showing that in patients with FL and clinical signs of HT, PET/CT has proven useful to identify transformed lymph nodes in patients with clinical signs and symptoms of HT, with SUVmax of transformed lymph nodes significantly higher than for nontransformed lymph nodes [16, 66]. Due to high variability of SUV measurements and PET/CT acquisitions, the standard deviation of SUVmax is high, which renders it difficult to define a threshold for HT [68]. In addition, overreliance on SUV in asymptomatic or low-risk FL may expose patients to unnecessary biopsies and treatment, since the overall prevalence of HT (and consequently the positive predictive value of PET/CT) is low in the absence of clinical symptoms of transformation [12, 13, 24]. However, a study on 38 transformed NHL, 23 of which were FL, showed that a SUVmax of 14 had a positive predictive value of 93.9% and a negative predictive value of 95.9% [66]. A study on 90 patients with CLL showed a significantly higher median SUVmax in patients with Richter syndrome, with different values if patients transformed to DLBCL or HL. Median SUVmax in patients with DLBCL was 14.6 in cases with DLBCL and 7 in cases with HL. Extranodal involvement was observed in 5 of 17 transformed cases, with splenic, gastric, skin, and tonsil lesions [69]. Regarding MZL, a study on 167 patients with MALT lymphoma treated with radiotherapy showed transformation in 7 cases (4%), in 5 cases at extranodal sites. In all cases, transformation occurred in sites different from the site of presentation [70]. In a study on 35 patients with WM/LPL, extranodal transformation was seen on PET/CT as a FDG-avid lesion in the bowel in 1 patient [54]. A small study compared 6 patients with NLPHL with its transformed counterpart, the TCRBCL showing that the average SUVmax was 6.9 in NLPHL and 16.6 in TCRBCL [71].

### Differential diagnosis

#### Progression

Differentiating progression of indolent lymphoma from TIL can be challenging, especially in the case of progression from low- to high-grade indolent lymphoma, as there is wide overlap between clinical, histologic, and molecular characteristics of progressed and transformed lymphoma [72–74]. On imaging, these can be indistinguishable, with similar imaging characteristics, and histologic confirmation should be obtained to differentiate the two entities, whenever possible (Fig. 1).

#### Recurrence

Due to the high recurrence rate of indolent lymphomas, the possibility of recurrence of disease after initial response should be always considered when patients present with increased adenopathy or increased extranodal disease on restaging scans. To differentiate recurrence from HT, the clinical presentation can be helpful: elevated LDH, new B or systemic symptoms are most likely associated with HT [12]. On imaging, diffuse mild increase in size of multiple lymph nodes, as well as areas with only mild increase in FDG-avidity, is most likely to be associated with recurrent indolent lymphoma [29].

#### Secondary malignancy

**Fig. 8:**
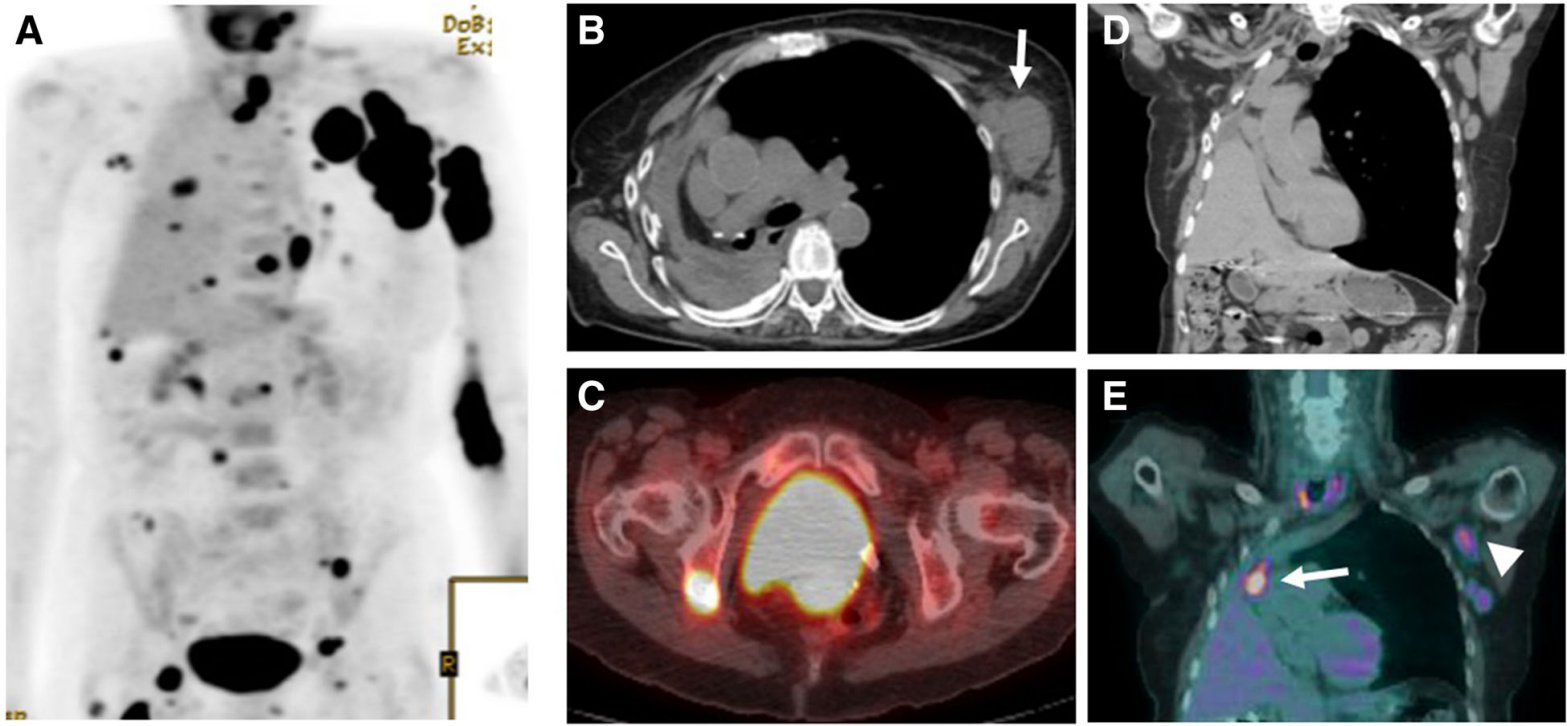
A 71-year-old woman with history of non-small cell lung cancer treated with pneumonectomy and erlotinib and grade I follicular lymphoma treated with chemotherapy presenting with new left arm swelling and a left axillary mass. **a** Coronal MIP reconstructed PET image shows multiple FDG avid left axillary adenopathy with SUVmax of 33.4, and scattered FDG avid foci in the left arm, within the chest and abdomen. **b** Axial CT image showed a large axillary adenopathy (arrow). **c** PET-CT fused axial image shows an area of FDG uptake within the left ischium. The left axillary adenopathy and the ischiatic lesion were biopsied and were consistent with large B cell lymphoma. **d** Coronal reconstructed CT image acquired during portal venous phase and (**e**) coronal MIP reconstructed PET image acquired 6 months before the onset of symptoms showed mild FDG uptake in the primary lung mass (arrow) and left axillary lymph nodes (arrowhead), which were thought to be consistent with metastatic spread of lung cancer

Patients with lymphoma are inherently at risk of secondary tumors due to the status of immunosuppression, and patients with history of cancer can develop lymphomas. Differentiating HT from tumor progression can be difficult, especially in cases of tumors which tend to metastasize to lymph nodes. Specific imaging features of the primary cancer, as well as concordance between primary tumor growth and nodal progression, or presence of new sites of metastasis, can be helpful to differentiate tumor progression from HT (Fig. 8).

### Imaging approach to transformation

**Fig. 9:**
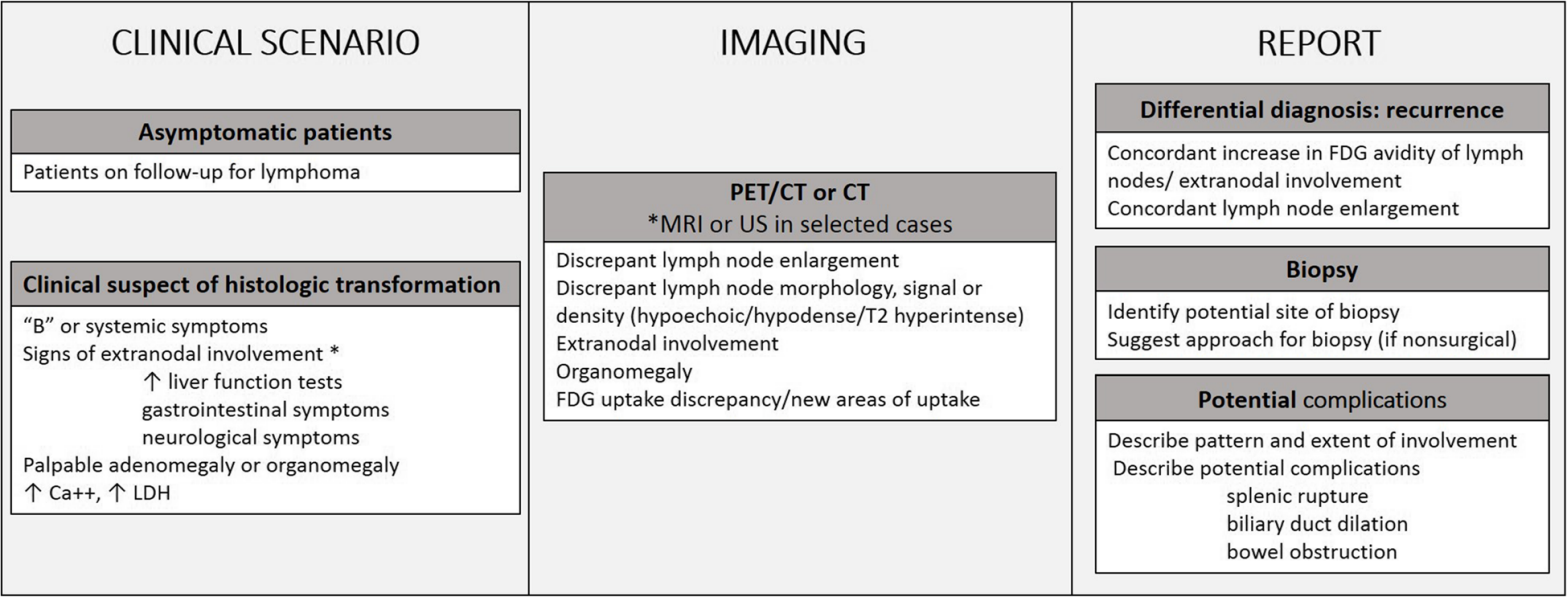
Imaging algorithm for management of suspected histologic transformation

An imaging algorithm for management of suspected histologic transformation is presented in Fig. 9.

#### Clinical scenario

Patients with HT could present to the radiologist in two ways: as asymptomatic patients undergoing surveillance for lymphoma or CLL with evidence of HT on imaging, or patients with clinical signs or symptoms suspicious for HT, including systemic signs, such as fatigue, infections or bleeding, “B” symptoms (fever, profuse night sweats, and unexplained weight loss), adenopathy or organomegaly, and increased LDH or hypercalcemia.

#### Imaging evaluation

Asymptomatic patients are generally imaged with CT or PET/CT. Symptomatic patients can undergo PET/CT if the primary lymphoma is known to be FDG avid; CT if the disease is not FDG avid, to evaluate imaging characteristic of nodal disease or extranodal involvement of disease or in cases of suspected emergent conditions (splenic rupture, bowel obstruction). MRI should be reserved in cases of potential extranodal involvement, such as bone marrow, liver, kidney, central nervous system, or soft tissue involvement. Ultrasound may be useful to study morphology and sonographic characteristics of superficial lymph nodes, as a first line imaging method to study focal extranodal involvement or to guide biopsy (Fig. 2). Once PET/CT has been obtained, images should be checked for presence of discrepancy in FDG avidity within the nodal groups or extranodal sites of disease, evidence of new or increased organomegaly and whenever possible, presence of decreased density of lymph nodes. On CT, US, and MR, presence of discrepant nodal enlargement, organomegaly, or morphologic and signal characteristics of HT, such as decreased lymph node density, heterogeneous echogenicity, or increased T2-hyperintensity for nodal disease, and typical imaging features, mentioned earlier in the paper, for extranodal involvement should raise suspicion for HT.

#### Description of findings

In every case and for every imaging modality, recurrent disease and development of secondary tumors should be ruled out, carefully evaluating the presence of concordant increase in nodal size or FDG avidity, extranodal involvement such as breast lesions and lung nodules. In addition, if HT is suspected on imaging, potential site of HT and approach for biopsy should be described when appropriate. Other potential complications should also be looked for and reported, including splenomegaly with impending splenic rupture, bowel obstruction, hydronephrosis, fractures, and bile duct dilation. It should be noted, however, that the likelihood of TIL in asymptomatic patients is low. Therefore, caution is necessary when raising concern of HT in these cases, as it may expose patients to unnecessary interventions and excess risks [13]. Finally, since indolent lymphomas are composed of multiple subpopulations with distinct mutations, multiple transformations can occur during the life of any patient with low-grade disease, sometimes simultaneously [11].

## Conclusion

Histologic transformation represents a critical point in the natural history of indolent lymphoma, with dramatic changes in patient prognosis and treatment. Knowing the most common types of indolent lymphoma and the natural history, biology, clinical, and imaging presentation of HT will help radiologists understand their role in patient management. Radiologists should be able to recognize signs of transformation, to identify a site for potential biopsy and to recognize mimickers or complications of HT, with an impact for patient prognosis.
